# The awareness of the Jordanian population about OTC medications: A cross‐sectional study

**DOI:** 10.1002/prp2.553

**Published:** 2019-12-27

**Authors:** Esra’ Taybeh, Zina Al‐Alami, Mervat Alsous, Mai Rizik, Zakaria Alkhateeb

**Affiliations:** ^1^ Department of Applied Pharmaceutical Sciences School of Pharmacy Isra University Amman Jordan; ^2^ Department of Medical Laboratory Sciences Faculty of Allied Medical Sciences Al‐Ahliyya Amman University Amman Jordan; ^3^ Department of Pharmacy Practice Faculty of Pharmacy Yarmouk University Irbid Jordan; ^4^ Department of Pharmacy Faculty of Pharmacy Amman Arab University Amman Jordan

**Keywords:** awareness, Jordan, knowledge, over‐the‐counter, patient information leaflet

## Abstract

Due to the shortage of literature related to the safe use of over‐the‐counter (OTC) products by patients worldwide, the aim of this study was to evaluate people's knowledge and attitudes regarding the use of OTC products in Jordan. Using an internet‐based questionnaire mainly spread through social media platforms, a descriptive cross‐sectional study was conducted with Jordanian candidates who consume OTC products. A total of 274 OTC product users answered the survey questions. The results showed that analgesics were the most commonly used OTC products among the participants (50.4%). The majority used the OTC products only as needed rather than on a regular basis. Only 42.4% of the participants sought a pharmacist's help in determining the dose of the OTC medicine. Most of the participants were very interested in reading a patient information leaflet (80.3%) and the side effects and contraindications (89.5%). The majority of participants agreed that antibiotics have to be prescribed (68.5%), and anti‐allergy medications should not be used as sleep aid medications (75.0%). About 53.4% thought that OTCs are sometimes enough to treat their health conditions without the need to follow‐up with a physician. A chi‐square analysis showed an association between gender, age, educational level and having a family member in the medical field and OTC products knowledge among Jordanians. Females, for example, were more interested in reading leaflet, checking production and expiry dates, knowing adverse effects, and appropriate storage conditions (*P* < .001, 0.022, 0.003, 0.007, respectively). We concluded that a good level of knowledge on the use of OTC products among the study population was identified in the present study.

**Abbreviations**
OTC: over‐the‐counter
PIL: patient information leaflet
Gps: general practitioners

## 1 INTRODUCTION

Over‐the‐counter (OTC) products include nonprescribed medications such as analgesics, cough and cold medicines, and anti‐fungal medications.^1^, ^2^ OTC medications are considered an important element of health care in Jordan 3 and the use of OTC products has steadily increased in the pharmaceutical market.^4^

Since OTC products are dispensed without a prescription, they are perceived by the public as a safer medicine compared to the prescribed ones.^5^, ^6^, ^7^ This perception has led patients to diagnose their own health and use inappropriate self‐medication.^8^ Misuse or abuse of OTC products by overusing a single agent or using too many different drugs to treat serious diseases 9 has led to misdiagnoses,^10^, ^11^ masking of serious conditions,^12^ addiction and dependency,^13^ kidney, liver, or gastric damage,^14^ and other health problems. Jordanians use OTC products, and the potential for abuse or misuse does exist.^15^, ^16^, ^17^

There are relatively few studies in the literature related to the safe use of OTC products among patients in Jordan. Previous studies in Jordan looked at the extent of OTC medication use and the reasons behind the prevalent use of OTC medications 15, 16, 17, 18; however, the recent studies have not evaluated the information about suspected abuse or misuse of OTC medication.

## 2 MATERIALS AND METHODS

A descriptive cross‐sectional study was conducted in February 2017. Jordanians were the source population for this study. According to the Internet World State in 2016, 73.6% of Jordanian people used the Internet. The sample size was calculated based on this percentage and was estimated at 269 participants. Patients aged 19 years and above, who used any OTC products and were willing to participate were considered for the study population.

The data were collected using an Internet‐based questionnaire after gaining ethical approval (November 1, 2016) from Isra University. The questionnaire was designed after a review of the relevant literature and written in Arabic.^16^, ^17^, ^18^, ^19^, ^20^, ^21^ Two independent faculty members at Isra University reviewed the questionnaire for its content and the questionnaire draft was revised based on their feedback. The survey was generated using “Google Forms” online survey platform. The survey had a cover page explaining the nature and purpose of the study. An informed consent form was obtained from each participant prior to the data collection. The questionnaire comprised 24 questions and it was divided into two parts: demographic data and questions on the use of OTC products. The latter part of the questionnaire covered three themes: the prevalence and types of OTC products used, the knowledge about the use of OTC products, and the satisfaction with OTC products used. The question and answer types varied from yes or no to drop‐down list answers. The survey was piloted among known OTC product users (n = 4). This involved completing the survey using different computers at different locations.

The link to the survey was distributed via Facebook platform which might not obtain the perceptions of people who do not use social media or do not have access or rarely use the internet. Therefore, the survey was also promoted through other possible data collection strategies (ie, telephone applications such as WhatsApp). To make sure our sample was random, the message containing the link was anonymously spread to different groups of social media users.

The SPSS software (version 21) was used for data analysis. Continuous data were reported as mean ± SD. The sociodemographic characteristics and descriptive statistics of participants are presented as frequency (percentages). A chi‐square test was conducted to explore the association between different categorical variables and OTC medication knowledge and attitudes. A confidence interval of 95% (*P* < .05) was applied to represent the statistical significance of the results. The level of significance was assigned as 5%.

## 3 RESULTS

### 3.1 Characteristics of the participants

A total number of 274 participants clicked on the link of the survey, but only 238 questionnaires were completely filled and included in the study analysis. Table 1 shows participant demographics.

**Table 1:** Demographic characteristics of participants n = 238

Parameter	N (%)
Age (years)	
19‐30	87 (36.6)
31‐40	92 (38.7)
41‐50	37 (15.5)
Over 50	22 (9.2)
Gender	
Male	46 (19.3)
Female	192 (80.7)
Nationality	
Jordanian	210 (88.2)
Other	28 (11.8)
Educational level*	
Less than secondary school	2 (0.8)
Secondary school	15 (6.3)
Community college‐medical	9 (3.9)
BSC medical	24 (10.1)
BSC nonmedical	54 (22.7)
Higher education medical	86 (36.1)
Higher education nonmedical	26 (10.9)
	22(9.2)
Family member in medical field	
Yes	194 (81.5)
No	44 (18.5)

* Higher education means Masters or PhD degrees.

### 3.2 The use of OTC products

**Figure 1:**
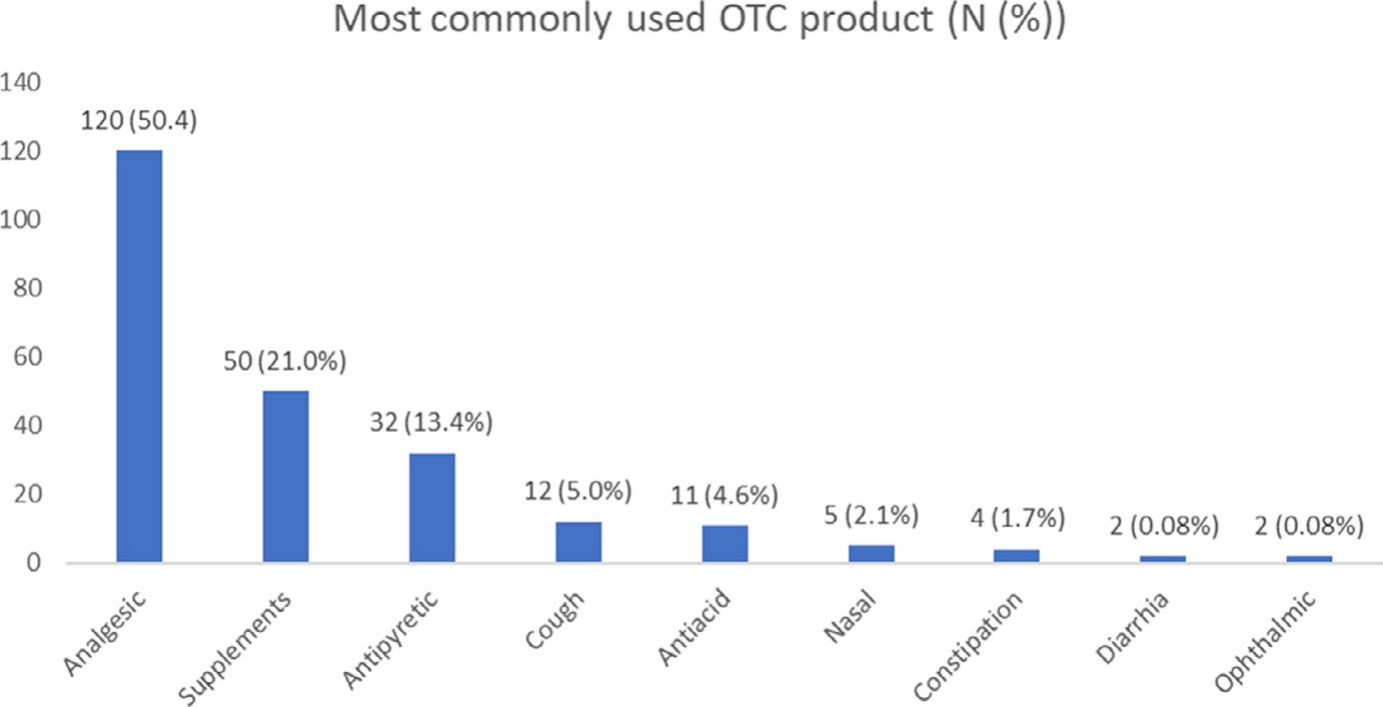
Commonly used OTC products among the participants

It was found that 23.9% of the participants use OTC products daily, 18.5% use them weekly, 16.4% use them monthly, and only 7.1% use them every 2 weeks. The rest (34.0%) use OTC products only as needed. Analgesics were the most commonly used OTC products among the participants (50.4%) followed by supplements (like vitamins and minerals) and antipyretics. Other categories of OTC products used were cough syrups, anti‐acids, nasal drops and sprays to reduce nasal congestion and a runny nose, constipation medications, diarrheal medications, and ophthalmic drops and ointments (Figure 1).

### 3.3 Information about OTC products

Only 39.9% of the participants considered physicians as the major source of information about OTC products, 34.9% sought advice from pharmacists, the other 25.2% depended on the consultation of friends, the media (newspapers, television, and radio), social media, and nurses, successively.

Most of the participants (89.5%) were interested in knowing the side effects and contraindications, and 80.3% were interested in reading the patient information leaflet, with females showing the greatest interest (*P* < .003). Besides that, 75.6% showed care regarding the appropriate storage conditions of OTC products, with female patients and participants who had family members working in the medical field showing more interest in reading about proper storage conditions (*P* < .007). About 37.0% of the participants knew the active ingredients of the OTC products they used.

The majority of participants (90.0%), mostly the females (*P* < .022), showed a great interest in checking the production and expiry dates (Table 2).

**Table 2:** Patients information about OTC products and factors affecting OTC products knowledge and use among Jordanians (n = 238)

Questions	Frequency (%)	N (%)	*P* value			
			Age	Gender	Education	Family Member
Most common used OTC medication	Analgesics	120 (50. 4)	0.097	0.101	0.529	0.444
	Supplements	50 (21.0)				
	Antipyretics	32 (13.4)				
	Cough	12 (5.0)				
	Anti‐acid	11 (4.6)				
	Nasal	5 (2.1)				
	Constipation	4 (1.7)				
	Diarrhea	2 (0.8)				
	Ophthalmic	2 (0.8)				
How often do you use the medications listed above?	Daily	57 (23.9.)	0.361	0.215	0.741	0.149
	Weekly	44 (18.5)				
	Monthly	39 (16.4)				
	Every 2 weeks	17 (7.1)				
	PRN	81 (3.4)				
What is your primary source of information about the medications listed above	Physician	95 (39.9)	0.503	0.745	0.411	0.136
	Pharmacist	83 (34.9)				
	Friends	23 (9.7)				
	Media	22 (9.2)				
	Social Media	11 (4.6)				
	Nurse	4 (1.7)				
Do you think antibiotics are prescription drugs?	Yes	163 (68.5)	0.478	0.111	0.011*	0.961
	No	75 (31.5)				
Do you repeat the use of eye drops or ointment from the same package after more than 1‐month postopening?	Yes	23 (9.7)	0.048*	0.401	0.332	0.915
	No	161 (67.6)				
	Sometimes	16 (6.7)				
	Never	38 (16.0)				
Do you repeat the use of nasal drops from the same packaging after more than a month after opening?	Yes	48 (20.2)	0.188	0.414	0.570	0.972
	No	23 (51.7)				
	Sometimes	24 (10.1)				
	Never	43 (18.1)				
Do you use cough medications after more than a month after opening them (solutions and syrups)	Yes	88	0.466	0.570	0.473	0.553
	No	87 (36.6)				
	Sometimes	42 (17.6)				
	Never	21 (8.8)				
Do you use anti‐allergy medications as a sleep aid medication?	Yes	8 (3.4)	0.097	0.563	0.006*	0.547
	No	177 (74.4)				
	Sometimes	22 (9.2)				
	Never	31 (13.0)				
Do you check production and expiry dates of OTC medications?	Yes	214 (89.9)	0.683	0.022*	0.132	0.395
	No	12 (5.0)				
	Sometimes	12 (5.0)				
Are you interested in reading the medicine leaflet of over‐the‐counter medications?	Yes	191 (80.3)	0.079	<0.001*	0.699	0.771
	No	13 (5.5)				
	Sometimes	34 (14.3)				
Do you like to read the side effects and contraindications of medications from their leaflets?	Yes	213 (89.5)	0.255	0.003*	0.593	0.888
	No	8 (3.4)				
	Sometimes	17 (7.1)				
Do you like to know from the drug leaflet the appropriate conditions for medications storage?	Yes	180	0.115	0.007*	0.874	0.031*
	No	23 (9.7)				
	Sometimes	35 (14.7)				
Who will help you in the dosage of the drug if it is without a prescription?	Pharmacist	101	0.927	0.193	0.767	0.097
	Leaflet	76 (31.9)				
	Physician	32 (13.4)				
	Myself	22 (9.2)				
	Friends	6 (2.5)				
	Nurse	1 (0.4)				
Do you tell your doctor about your prescription medications without even asking you when you talk about your illness?	Yes	173 (72.7)	0.001*	0.068	0.189	0.632
	No	33 (13.9)				
	Sometimes	32 (13.4)				
Do you know the name of the active ingredient in the OTC medication?	Yes	88 (37.0)	0.019*	0.019*	<0.001*	0.115
	No	87 (36.5)				
	Sometimes	63 (26.5)				
Do you think that your OTC medications are enough to treat you, and do not require visiting your doctor after taking them?	Yes	69 (29.0)	0.031*	0.005*	0.367	0.479
	No	42 (17.6)				
	Sometimes	127 (53.4)				
Did you have side effects as a result of using a prescription drug?	Yes	28	0.183	0.849	0.759	0.061
	No	193 (81.1)				
	Do not know	17 (7.1)				

* *P* <.05.

A percentage of 42.4% sought a pharmacist's help to determine the appropriate dose. Others depended on the patient information leaflet, consulted a physician, or depended on their own information, and others asked friends and nurses. Additionally, three quarters of the participants inform their physician about the OTC products they take, even without being asked.

### 3.4 Knowledge about OTC products

Some participants (68.5%), especially those with higher education (holding a Master or PhD degree; *P* < .011), reported that they think that antibiotics have to be prescribed and never dispensed without prescriptions. Moreover, the majority of participants agreed that nasal drops (51.7%) or eye drops/ointments (67.6%) should not be used after the medicine package was open more than 1 month. This latter finding was affected by the user's age with those aged 41‐50 years being more aware (*P* < .048). On the other hand, participants’ responses were evenly distributed regarding their use of cough medications (solutions and syrups) a month after opening. This finding needs further investigation about the different types of cough medications, their shelf‐lives, efficacy, and safety. Around 75.0% of participants reported that they do not use anti‐allergy medications as a sleep aid medication. Using anti‐allergic medications as a sleep aid was inversely related with the educational level (*P* < .006; Table 2).

### 3.5 Patient satisfaction after OTC product use

Participants who claimed that they suffered from side effects as a result of using OTC products were only 11.8% whereas 7.1% were not aware whether they went through any side effects at all. Additionally, 53.4% of the participants thought that OTC products are sometimes enough in treating their conditions without the need to follow‐up with physician while only 29.0% believed that OTC products are satisfying and there is no need to consult a physician (Table 2).

## 4 DISCUSSION

According to our results, the knowledge and attitudes of Jordanians regarding OTC was overall good in most aspects of the study. Analgesics/antipyretics were the most commonly used self‐medications among OTC products, which was in agreement with previous studies.^19^, ^20^, ^21^, ^22^ This can be attributed to the common use of such drugs in treating minor and uncomplicated illnesses such as fever, headache, and pain.^23^ Beside analgesics, supplements were the second mostly used OTC products, which is similar to what was reported in different countries such as Malaysia and the United States.^24^, ^25^ This can be explained by the fact that people perceive supplements and vitamins as safe, effective, and necessary additions for good health.

The majority of participants reported that physicians and pharmacists were the main sources of information regarding OTC products. Previous studies also reported consistent results where pharmacists appeared to be the main source of information.^26^, ^27^, ^28^, ^29^ This highlights the crucial role of the pharmacist in promoting awareness regarding the importance of the safety and the appropriate use of OTC products.

Many studies have shown that most patients store their medications improperly at their home which may lead to undesirable side effects or unintentional risks. Patients keep medications because they do not want to waste them, they do not know how to read and check the expiry date, or they do not know a proper or safe way to dispose of them.^30^, ^31^, ^32^ Fortunately, around 90.0% of our participants check the production and expiry date of OTC products, which reflects a good level of awareness of medication safety. A previous Swedish study reported that 54.0% of their respondents always or usually check the expiration date of their OTC product, while only 12.0% said they never do.^33^

Our results show that the awareness of our respondents generally, and females particularly, about OTC products was highly associated with checking the expiry dates. This might be because women are known for being the home medical caregiver which is a common pattern in many parts of the world.

A patient information leaflet (PIL) is intended to provide patients with written information about the medication. Its main purpose is to provide patients with the necessary information about their medication in terms of its administration, precautions, and potential side effects.^34^ In our study, gender was highly associated with reading OTC product leaflets including side effects, contraindications, and proper storage conditions (*P* < .05). These findings were in line with what was found in Palestine where gender was the only associated factor with reading the PIL. Besides, reading the PIL was significantly associated with the female gender (*P* = .047).^35^ This could be due to the general positive attitude of women toward medication and their responsibility of managing the household.

However, PILs cannot replace the judgment of general practitioners (GPs) and patients usually have much more confidence in their GP's knowledge. Our respondents were highly alert in terms of communicating with their physicians about their medications. However, previous reports suggested that many patients taking nonprescription medication do not tend to disclose the use of these medications to their physician.^36^, ^37^ This could be explained by the lack of good quality and adequate quantity of health providers, either doctors or pharmacists, counseling on the appropriate use of both prescribed and nonprescribed medications. Also, time constraints during doctors’ visits may play a part in patients not disclosing all their relevant medical information.

It is necessary for patients to recognize the active ingredients to avoid many undesirable side effects, potential interactions, and unintentional overdoses. Responses about knowing the active ingredients of OTC products in our study widely varied with only 37% of the respondents replying "yes" to the question about the name of OTC ingredients. Gender, age, and education level were all determinants for recognizing the active ingredients in OTC products in our study (*P* < .05). Most reports agree that people with low literacy are more susceptible to unsafe medication use, adherence to medication,^38^, ^39^ and over dosing.^40^ Adequate literacy was associated with more than 10 times the odds of choosing the appropriate medication along with the use of active ingredients as part of their rationale.^41^

In our study, it was clear that the respondents had some level of awareness in regards to antibiotics being classified as prescribed medications. Around 70.0% agreed that antibiotics are prescribed medications and not OTC products. Education was significantly associated with our respondents’ perception in this regard. Respondents with a higher education (holding a master of a PhD degree) were identified as significantly associated with knowing that antibiotics are prescribed medications (*P* < .05). The lack of knowledge among those with lower education has been already described in other studies conducted in different countries.^42^, ^43^, ^44^ According to a previous study conducted in Jordan, people knowledgeable about antibiotics and those with a high education reported using antibiotics without a physician's consultation and keeping left‐over antibiotics. From the same study, 31.5% of respondents did not know that antibiotics are prescribed medicines only. Moreover, it was reported that people in Jordan had an inadequate knowledge about antibiotic use, the efficacy of antibiotics, and the risk of antibiotic. Their results showed that people were clearly confused regarding whether antibiotics are effective against bacteria and viruses.^45^ These findings shed light on the importance of developing a comprehensive and multifaceted intervention including a focused and comprehensive education involving definitions, antibiotic uses, and clarified misconceptions and myths surrounding antibiotic use to reduce public self‐medication, reconstruct their expectations regarding antibiotics, and raise awareness about antibiotic resistance. In addition, and more importantly, some radical changes need to be adopted and more strict regulation enforced to reduce the common practice of obtaining antibiotics with prescriptions by either obtaining them over the phone without actual physical examination or via community pharmacies.

Responses regarding efficacy of OTC products in treating illnesses without consulting a physician varied among our respondents. Most patients (69.0%) responded positively regarding the efficacy of OTC in treating their ailments. However, OTC products and self‐medication can alleviate minor illnesses and thus forgo a medical consult and reduce pressure on the supply of medical services especially in less developed countries.^46^

The present work answered a series of related questions regarding public knowledge, awareness, and attitudes about OTC medications which represent one important aspect in the study of rational drug use. The findings strongly supported the importance of the health care role in patient's education where future health care plans should emphasize the pharmacy section's role to better educate patients about the correct and safe use of OTC products. Our study adds to what has been previously acknowledged globally but we believe that, to the best of our knowledge, the items covered by our study is among the first few local studies that address patients' attitudes toward OTC medications.

A low sample size but one comparable to other online survey studies and much higher percentage of female participation were the main limitations of this study. In addition, the use of an online survey limits the generalizability of our results to patients who have access to the internet and the use of social media, which is not the case for elderly and other patients of low income. Finally, asking the patients about their experience of adverse effects after using OTC medications recalls bias which might affect the accuracy of the data. Furthermore, issues related to the duration of OTC and drug interactions were beyond the scope of the present study.

## 5 CONCLUSIONS

A good level of knowledge about the use of OTC products among the study population was identified in the present study in terms of reading the patient information leaflet, knowing the active ingredients, side effects, and contraindications, and in knowing the misuse/abuse practices of OTC products. Since a high prevalence of OTC product use is evident, OTC product users should also be encouraged to regularly consult health care providers in the case of lack of knowledge.

## DISCLOSURE

We declare that there is no conflict of interest.

## AUTHOR CONTRIBUTIONS

Esra’ Taybeh and Zina Al‐Alami conceived and designed the research, performed the study, and wrote and revised the manuscript. Mervat Alsous performed data analysis, wrote and revised the manuscript. Mai Rizik contributed in manuscript writing. Zakaria Alkhateeb designed the research tool and collected the data.
