# Supplementary material for: The awareness of the Jordanian population about OTC medications: A cross‐sectional study
Source: Pharmacol Res Perspect. 2019 Dec 27;8(1):e00553. doi: 10.1002/prp2.553 (PMC6934420; doi:10.1002/prp2.553)
Supplement: Supplementary file 1 [file PRP2-8-e00553-s001.docx]

**Supplementary material**

Table 1: Association between knowing that antibiotic is not an OTC medication and participants education

| Educational Level |  | Do you think antibiotics are prescription drugs? | | Total | Pearson Chi-Square | sig |
| --- | --- | --- | --- | --- | --- | --- |
|  |  | Yes | No |  |  |  |
| Less than secondary school | Count | 2 | 0 | 2 | 18.126 | .011 |
|  | % of Total | .8% | .0% | .8% |  |  |
| Secondary school | Count | 8 | 7 | 15 |  |  |
|  | % of Total | 3.4% | 2.9% | 6.3% |  |  |
| Community collge medical field | Count | 5 | 4 | 9 |  |  |
|  | % of Total | 2.1% | 1.7% | 3.8% |  |  |
| Community collge non-medical field | Count | 9 | 15 | 24 |  |  |
|  | % of Total | 3.8% | 6.3% | 10.1% |  |  |
| BSc - medical field | Count | 14 | 40 | 54 |  |  |
|  | % of Total | 5.9% | 16.8% | 22.7% |  |  |
| BSc non- medical field | Count | 28 | 58 | 86 |  |  |
|  | % of Total | 11.8% | 24.4% | 36.1% |  |  |
| Higher education - medical field | Count | 2 | 24 | 26 |  |  |
|  | % of Total | .8% | 10.1% | 10.9% |  |  |
| Higher education - non-medical field | Count | 7 | 15 | 22 |  |  |
|  | % of Total | 2.9% | 6.3% | 9.2% |  |  |

Table 2: Association between using OTC eye preparations and participants age

|  | | | Do you repeat the use of eye drops or ointment from the same package after more than one-month post opening? | | | | Total | Pearson Chi-Square | sig |
| --- | --- | --- | --- | --- | --- | --- | --- | --- | --- |
|  |  |  | Yes | No | Sometimes | Never used |  |  |  |
| Age | 19-30 | Count | 5 | 53 | 6 | 23 | 87 | 17.054 | .048 |
|  |  | % of Total | 2.1% | 22.3% | 2.5% | 9.7% | 36.6% |  |  |
|  | 31-40 | Count | 12 | 66 | 6 | 8 | 92 |  |  |
|  |  | % of Total | 5.0% | 27.7% | 2.5% | 3.4% | 38.7% |  |  |
|  | 41-50 | Count | 3 | 25 | 2 | 7 | 37 |  |  |
|  |  | % of Total | 1.3% | 10.5% | .8% | 2.9% | 15.5% |  |  |
|  | older than 50 | Count | 3 | 17 | 2 | 0 | 22 |  |  |
|  |  | % of Total | 1.3% | 7.1% | .8% | .0% | 9.2% |  |  |

Table 3: Association between using anti-allergy OTC medications for sleep and participants education

| Educational Level |  | Do you use anti-allergy medications as a sleeping aid medication? | | | | Total | Pearson Chi-Square | sig |
| --- | --- | --- | --- | --- | --- | --- | --- | --- |
|  |  | Yes | No |  |  |  |  |  |
| Less than secondary school | Count | 0 | 1 | 1 | 0 | 2 | 40.703^a^ | 0.006 |
|  | % of Total | .0% | .4% | .4% | .0% | .8% |  |  |
| Secondary school | Count | 3 | 7 | 1 | 4 | 15 |  |  |
|  | % of Total | 1.3% | 2.9% | .4% | 1.7% | 6.3% |  |  |
| Community collge medical field | Count | 1 | 7 | 1 | 0 | 9 |  |  |
|  | % of Total | .4% | 2.9% | .4% | .0% | 3.8% |  |  |
| Community collge non-medical field | Count | 0 | 15 | 2 | 7 | 24 |  |  |
|  | % of Total | .0% | 6.3% | .8% | 2.9% | 10.1% |  |  |
| BSc - medical field | Count | 1 | 37 | 8 | 8 | 54 |  |  |
|  | % of Total | .4% | 15.5% | 3.4% | 3.4% | 22.7% |  |  |
| BSc non- medical field | Count | 1 | 67 | 8 | 10 | 86 |  |  |
|  | % of Total | .4% | 28.2% | 3.4% | 4.2% | 36.1% |  |  |
| Higher education - medical field | Count | 1 | 24 | 0 | 1 | 26 |  |  |
|  | % of Total | .4% | 10.1% | .0% | .4% | 10.9% |  |  |
| Higher education - non-medical field | Count | 1 | 19 | 1 | 1 | 22 |  |  |
|  | % of Total | .4% | 8.0% | .4% | .4% | 9.2% |  |  |

Table 4: Association between checking production and expiry dates of an OTC medication and participants gender

|  | | | Do you check production and expiry dates of OTC medications? | | | Total | Pearson Chi-Square | sig |
| --- | --- | --- | --- | --- | --- | --- | --- | --- |
|  |  |  | Yes | No | Sometimes |  |  |  |
| Gender | Male | Count | 38 | 6 | 2 | 46 | 7.634 | .022 |
|  |  | % of Total | 16.0% | 2.5% | .8% | 19.3% |  |  |
|  | Female | Count | 176 | 6 | 10 | 192 |  |  |
|  |  | % of Total | 73.9% | 2.5% | 4.2% | 80.7% |  |  |

Table 5: Association between reading leaflet of an OTC medication and participants gender

|  | | | Are you interested in reading the medicine leaflet of over-the-counter medications? | | | Total | Pearson Chi-Square | sig |
| --- | --- | --- | --- | --- | --- | --- | --- | --- |
|  |  |  | Yes | No | Sometimes |  |  |  |
| Gender | Male | Count | 28 | 10 | 8 | 46 | 30.712 | .000 |
|  |  | % of Total | 11.8% | 4.2% | 3.4% | 19.3% |  |  |
|  | Female | Count | 163 | 3 | 26 | 192 |  |  |
|  |  | % of Total | 68.5% | 1.3% | 10.9% | 80.7% |  |  |

Table 6: Association between reading adverse effects of an OTC medication and participants gender

|  | | | Do you like to read the side effects and contraindications of medications from their leaflets? | | | Total | Pearson Chi-Square | sig |
| --- | --- | --- | --- | --- | --- | --- | --- | --- |
|  |  |  | Yes | No | Sometimes |  |  |  |
| Gender | Male | Count | 40 | 5 | 1 | 46 | 11.575 | .003 |
|  |  | % of Total | 16.8% | 2.1% | .4% | 19.3% |  |  |
|  | Female | Count | 173 | 3 | 16 | 192 |  |  |
|  |  | % of Total | 72.7% | 1.3% | 6.7% | 80.7% |  |  |

Table 7: Association between reading storage conditions of an OTC medication and participants gender

|  | | | Do you like to know from the drug leaflet the appropriate conditions for medications storage? | | | Total | Pearson Chi-Square | sig |
| --- | --- | --- | --- | --- | --- | --- | --- | --- |
|  |  |  | Yes | No | Sometimes |  |  |  |
| Gender | Male | Count | 29 | 10 | 7 | 46 | 9.808 | .007 |
|  |  | % of Total | 12.2% | 4.2% | 2.9% | 19.3% |  |  |
|  | Female | Count | 151 | 13 | 28 | 192 |  |  |
|  |  | % of Total | 63.4% | 5.5% | 11.8% | 80.7% |  |  |

Table 8: Association between reading storage conditions of an OTC medication and having a family member in medical field

|  | | | Do you like to know from the drug leaflet the appropriate conditions for medications storage? | | | Total | Pearson Chi-Square | sig |
| --- | --- | --- | --- | --- | --- | --- | --- | --- |
|  |  |  | Yes | No | Sometimes |  |  |  |
| Having a family member in medical field | Yes | Count | 140 | 21 | 33 | 194 | 6.919 | 0.031 |
|  |  | % of Total | 58.8% | 8.8% | 13.9% | 81.5% |  |  |
|  | No | Count | 40 | 2 | 2 | 44 |  |  |
|  |  | % of Total | 16.8% | .8% | .8% | 18.5% |  |  |
